# Supplementary material for: Care home resident identification: A comparison of address matching methods with Natural Language Processing
Source: PLoS One. 2024 Dec 5;19(12):e0309341. doi: 10.1371/journal.pone.0309341 (PMC11620595; doi:10.1371/journal.pone.0309341)
Supplement: S1 Appendix — (DOCX) [file pone.0309341.s001.docx]

**S1 Appendix: Filtering selection**

The filtering process determines conditions to be met before processing using simple rules and basic comparisons developed during initial data exploration. For example, lack of postcode match was found to be a good discarding indicator with a low number of false negatives. Filtering involves the application of four different conditions:

1. **CHname**: whether the patient's first address line contains a string related to a care home name such as "care home", "nursing home", or "residential home”. If so, the similarity is directly assigned to 0, which means this CHI address is labelled as a care home address. Otherwise, the process continues evaluating the following conditions.
2. **PCmatch**: checks if the patient and the care home postcodes are valid using a regular expression extracted from the guidance on bulk data transfer available in the Government information website of the United Kingdom [21] and if they match when both are transformed to lowercase, and spaces are removed.
3. **CHserv**: filters the care home by its type of service using a predefined list of target services ("*Older People*" and "*Physical and Sensory Impairment*" i.e., excluding services for people with learning disabilities, alcohol misuse or mental health).
4. **CHtown**: uses the town in the care home address and tries to find a string match in the different CHI address lines.

The pipeline continues to the next step only if the **PCmatch**, **CHserv**, and **CHtown** conditions are true, otherwise, the similarity is assigned to 1, which means the patient and care home addresses are not considered a match. Tables S1 and S2 present the performance of different combinations of filters using the Damerau-Levenshtein with 1-char-length n-gram to determine the best configuration for address and patient levels, respectively.

**Table S1: Results for different filtering approaches at the address level in the validation set to validate the best configuration calculated using the Damerau-Levenshtein distance with 1-char-length n-gram. Bold numbers show the best result for each metric in each health board and population.**

**Table S2: Results for different filtering approaches at the patient level in the validation set to validate the best configuration calculated using the Damerau-Levenshtein distance with 1-char-length n-gram. Bold numbers show the best result for each metric in each health board and population.**
