# Supplementary material for: Care home resident identification: A comparison of address matching methods with Natural Language Processing
Source: PLoS One. 2024 Dec 5;19(12):e0309341. doi: 10.1371/journal.pone.0309341 (PMC11620595; doi:10.1371/journal.pone.0309341)
Supplement: S2 Appendix — (DOCX) [file pone.0309341.s002.docx]

**S2 Appendix: Tuning n-gram**

The n-gram length is an important hyper-parameter to be tuned because it determines the number of characters (chars) to be compared by the edit distance and to create the vector space model. Thus, it was validated for the string and vector representations using the conditions **PCmatch**, **CHserv**, and **CHname** as filtering processes with the Damerau-Levenshtein and the Cosine distances, respectively. The 2-chars-length n-grams for the edit distance calculation got slightly better results than the 1-char-length, especially in the PPV metrics for the Tayside health board (see Tables S3 and S4 for address and patient levels, respectively). Similarly, the Cosine distance calculated with 1-char-length performs better for the Fife health board while the 2-chars-length achieves greater results in the Tayside addresses and patient level recognition (see Tables S5 and S6 for address and patient levels, respectively). In this case, the combination of 1 and 2 chars to form the vector representation for each address does not reach the performance of only using 1 or 2 chars. To summarize, according to the results, setting character n-grams to 2 achieves better results in Tayside while 1-grams obtain higher performance in Fife.

**Table S3: Results for different char n-gram lengths at the address level in the validation set to validate the best configuration calculated using the Damerau-Levenshtein distance. Bold numbers show the best result for each metric in each health board and population.**

**Table S4: Results for different char n-gram lengths at the patient level in the validation set to validate the best configuration calculated using the Damerau-Levenshtein distance. Bold numbers show the best result for each metric in each health board and population.**

**Table S5: Results for different char n-gram lengths at the address level in the validation set to validate the best configuration calculated using the Cosine distance. Bold numbers show the best result for each metric in each health board and population.**

**Table S6: Results for different char n-gram lengths at the patient level in the validation set to validate the best configuration calculated using the Cosine distance. Bold numbers show the best result for each metric in each health board and population.**
