# Supplementary material for: Care home resident identification: A comparison of address matching methods with Natural Language Processing
Source: PLoS One. 2024 Dec 5;19(12):e0309341. doi: 10.1371/journal.pone.0309341 (PMC11620595; doi:10.1371/journal.pone.0309341)
Supplement: S3 Appendix — (DOCX) [file pone.0309341.s003.docx]

**S3 Appendix: Edit distance**

Approximate string matching (also known as fuzzy string matching) is a group of methods that calculate the edit distance, which is the minimum number of changes required to transform one string into another. Contrary to exact string matching, these methods give a similarity score being low when the strings are similar. The following are the edit distances used for the experiments:

- **Levenshtein distance** [22] is a string metric that calculates the distance between two string inputs using the operations of insertion, deletion, and substitution.
- **Damerau–Levenshtein distance** [23] is a string metric that includes the transposition of two adjacent characters together with the insertion, deletion, and substitution operations.
- **Jaro distance** [24] considers only the minimum number of character transpositions as the operation to measure the edit distance.
- **Jaro-Winkler distance** [25] is a modification of the previous metric that gives more relevance to the prefixes with a predefined length of the strings.

To normalize the score given by these similarity metrics between 0 and 1 for the experiments, we divide the Levenshtein and Damerau–Levenshtein distances by the maximum length for both inputs. The edit distance computation calculates each operation character by character but, this can be generalized to $n$ characters (n-grams). This generalization outperforms the one-character edit distance because it takes more information about the context [26].
