# Supplementary material for: Care home resident identification: A comparison of address matching methods with Natural Language Processing
Source: PLoS One. 2024 Dec 5;19(12):e0309341. doi: 10.1371/journal.pone.0309341 (PMC11620595; doi:10.1371/journal.pone.0309341)
Supplement: S4 Appendix — (DOCX) [file pone.0309341.s004.docx]

**S4 Appendix: Vector space model**

Text documents can be transformed into numeric representations using vector space models. Each dimension in these vectors represents a value of each term in a dictionary. These values are counts of the occurrences for the terms presented in the document, while the remaining terms in the dictionary are 0.

The single characters of the alphabet from a to z and the numbers from 0 to 9 are considered the dictionary of terms for the addresses. In addition, we extended the vocabulary to a higher number of characters (n-grams), thus, we can increase the representation of each address using dictionaries with more than one character and concatenate vocabularies of different sizes to form a vector. For instance, the address line "89 Bay Street" is separated into *{"8":1, "9":1, " ":2, "t":3, "a":1, "y":1, "s":1, "r":1, "e":2}* using a single-character dictionary, into *{"89":1, "9 ":1, " t":1, "ba":1, "ay":1, "y ":1, " s":1, "st":1, "tr":1, "re":1, "ee":1, "es":1}* using a two-character dictionary, and the concatenation of both representations if single-character and two-character dictionaries are being used together.

Once each address is transformed into vector representations, spatial distance can be computed to measure the similarity between them. These are the distance functions applied to two numeric vectors:

- **City Block (Manhattan)** represents the absolute differences between a pair of vectors $u$ and $v$, defined as $\sum_{i} \left| \left( u_{i}-v_{i} \right) \right|$ and normalized with $L_{1}$-norm to be in the range 0,1.
- **Euclidean** represents the root of the square differences between a pair of vectors $u$ and $v$, defined as $\sqrt{\sum_{i} \left( u_{i}-v_{i} \right)^{2}}$, and normalized with $L_{2}$-norm to be in the range 0,1.
- **Cosine** measures the cosine of the angle between a pair of vectors $u$ and $v$. Calculated as $\frac{\sum_{i} u_{i}v_{i}}{\sqrt{\sum_{i} u_{i}^{2}}\sqrt{\sum_{i} v_{i}^{2}}}$.
- **Correlation** based on the Pearson correlation coefficient [27], measures the covariance of a pair of vectors $u$ and $v$ divided by the root of the multiplication of their square variances. Calculated as $\frac{\sum_{i} \left( u_{i}-u‾ \right)\left( v_{i}-v‾ \right)}{\sqrt{\sum_{i} \left( u_{i}-u‾ \right)^{2}}\sqrt{\sum_{i} \left( v_{i}-v‾ \right)^{2}}}$ being $u‾$ and $v‾$ the mean of the vectors $u$ and $v$, respectively, and divided by $2$ to be in the range 0,1.
- **Jensen-Shannon** is calculated using the Kullback-Leibler divergences ($D$) [28] with the probability vectors $p$ and $q$ to their pointwise mean $m$ as $\sqrt{\frac{D\left( p||m \right)+D\left( q||m \right)}{2}}$.
- **Canberra** [29] represents the absolute difference divided by the sum of the vectors $u$ and $v$, defined as $\sum_{i} \frac{\left| u_{i}-v_{i} \right|}{\left| u_{i} \right|+\left| v_{i} \right|}$.
- **Bray-Curtis** [30] represents the sum of the fractions between each absolute difference and the absolute additions of the vectors $u$ and $v$, defined as $\frac{\sum_{i} \left| u_{i}-v_{i} \right|}{\sum_{i} \left| u_{i}+v_{i} \right|}$.
