# Supplementary material for: Care home resident identification: A comparison of address matching methods with Natural Language Processing
Source: PLoS One. 2024 Dec 5;19(12):e0309341. doi: 10.1371/journal.pone.0309341 (PMC11620595; doi:10.1371/journal.pone.0309341)
Supplement: S5 Appendix — (DOCX) [file pone.0309341.s005.docx]

**S5 Appendix: Similarity score computation**

Once the address lines and the list of numbers are preprocessed for the patient and care home addresses, Algorithm 1 is applied to calculate the similarity score between both. This step determines the minimum similarity measure for a CHI address line to each line of a care home address using a predefined distance approach. In the case of the lists of numbers for CHI and care home addresses the scores are calculated by exact matching, being 0 if they are the same and 1 if they are different. The minimum similarity measures for each CHI address line and numbers to a care home address are averaged to get a score, which represents the final similarity between them.

**Algorithm 1: Calculate the similarity score 0,1 (being 0 when they are the same address) between a CHI address (*pAddr*) and a care home address (*chAddr*) with a predefined DISTANCE.**

*1:* **Input:** CHI address, *pAddr*, and a care home address, *chAddr*.

*2:* **for** *pLine* **in** *lines* contained in *pAddr* ***do***

*3:* **for** *chLine* **in** *lines* contained in *chAddr* ***do***

*4: distance_j_ ←* DISTANCE*(pLine, chLine)* (compute distance between address lines)

*5:* **end**

*6: line_simil_i_ ←* min*_j_(distance_j_)* (take the minimum distance)

*7:* **end**

*8:* **for** *pNum* **in** *numbers* contained in *pAddr* **do**

*9:* **for** *chNum* **in** *numbers* contained in *chAddr* **do**

*10: distance_j′_ ←* not*(pNum==chNum)* (check whether numbers are equals)

*11:* **end**

*12: line_simil_i′_ ←* min*_j′_(distance_j′_)* (take the minimum distance)

*13:* **end**

*14: similarity ← (∑_i_ line_simil_i_ + ∑_i′_ line_simil_i′_)/(i + i′)* (average and sum the minimum distance)

*15:* **Output:** *similarity*

The whole process is repeated over the official care home list. Thus, the final score to find the possible residence status is the minimum similarity measure between a CHI address and all the care home addresses. In the training process, a threshold was applied as a cut-off value to assign the care home resident status to those addresses with a score below the threshold. Figure S1 shows an example of how performance for PPV, sensitivity and F1 measures change when varying the similarity threshold value during the training phase. F1 is included in this research as an NLP standard metric to optimize PPV and sensitivity at the same time. It can be observed that the sensitivity is low at a low threshold because all addresses are recognized as non-residents, but PPV is high because addresses identified as care homes are essentially exact matches. With an increasing threshold, sensitivity increases addresses are matched to those of care homes, but PPV decreases.

**Figure S1: Behaviour of system performance measured in PPV (in red), sensitivity (in green) and F1 (in blue) using the training set with an increasing threshold for similarity score (0=exact match, 1=no similarity). The black dot represents the threshold that maximizes the F1 measure.**

The final threshold is chosen to maximize a measured value (F1 in the experiments) using the training set. Later, the performance for a specific distance approach is measured using the pre-trained threshold in the validation set. For the experiments, the CHI address dataset was divided into training and validation sets. For each health board (Tayside and Fife) overall and 65 and over populations, data sets are split into 70% for training and 30% for validation of the total sample, respectively. In each set, we ensured the same proportion of positive and negative instances, i.e., CHI addresses which were manually labelled as matching a care home address or not.

Detailed results of the performance in each health board are presented for the baseline methods in Tables S7 and S8, and for the proposed methods in Tables S9 and S10, both using the address level and patient level, respectively.

**Table S7: Baseline and state-of-the-art methods for detecting care home residents at the address level in the validation set. Cut-off values are in brackets for the Markov and Phonics scores. Bold numbers show the best result for each metric in each health board and population.**

**Table S8: Baseline and state-of-the-art methods for detecting care home residents at the patient level in the validation set. Cut-off values are in brackets for the Markov and Phonics scores. Bold numbers show the best result for each metric in each health board and population.**

**Table S9: Final results at the address level in the validation set using different measures of distance for string and vectors with the best configurations (2-char-length n-grams in both distances). The filtering process used was the PCmatch, CHserv, and CHname. Bold numbers show the best result for each metric in each health board and population.**

**Table S10: Final results at the patient level in the validation set using different measures of distance for string and vectors with the best configurations (2-char-length n-grams in both distances). The filtering process used was the PCmatch, CHserv, and CHname. Bold numbers show the best result for each metric in each health board and population.**
