# Supplementary material for: Care home resident identification: A comparison of address matching methods with Natural Language Processing
Source: PLoS One. 2024 Dec 5;19(12):e0309341. doi: 10.1371/journal.pone.0309341 (PMC11620595; doi:10.1371/journal.pone.0309341)
Supplement: S6 Appendix — (DOCX) [file pone.0309341.s006.docx]

**S6 Appendix: performance measures**

Performance was evaluated using Positive Predictive Value (PPV), sensitivity, Negative Predictive Value (NPV), specificity, and an F1 metric, which is the harmonic mean of PPV and sensitivity, all of which are defined using the numbers of True Positives (TP), True Negatives (TN), False Positives (FP) and False Negatives (FN) of the classifiers compared to gold-standard manual allocation as follows:

$PPV=\frac{TP}{TP+FP}$ (1)

$Sensitivity=\frac{TP}{TP+FN}$ (2)

$NPV=\frac{TN}{TN+FN}$ (3)

$Specificity=\frac{TN}{TN+FP}$ (4)

$F1=2\times\frac{PPV\times Sensitivity}{PPV+Sensitivity}$ (5)
