# Supplementary material for: Care home resident identification: A comparison of address matching methods with Natural Language Processing
Source: PLoS One. 2024 Dec 5;19(12):e0309341. doi: 10.1371/journal.pone.0309341 (PMC11620595; doi:10.1371/journal.pone.0309341)

PLOS ONE

Care home resident identification: A comparison of address matching methods with Natural Language Processing

--Manuscript Draft--

**Manuscript Number:**	PONE-D-23-42367
**Article Type:**	Research Article
**Full Title:**	Care home resident identification: A comparison of address matching methods with Natural Language Processing
**Short Title:**	Care home resident identification: A comparison of address matching methods with Natural Language Processing
**Corresponding Author:**	Víctor Suárez-Paniagua, Ph.D.<br>The University of Edinburgh<br>Edinburgh, UNITED KINGDOM
**Keywords:**	Care Home, Address Matching, Natural Language Processing, Similarity Measure, Older People
**Abstract:**	Background: Care home residents are a highly vulnerable group, but identifying care home residents in routine data is challenging. This study aimed to develop and validate Natural Language Processing (NLP) methods to identify care home residents from primary care address records.<br>Methods: The proposed system applies an NLP sequential filtering and preprocessing of text, then the calculation of similarity scores between general practice (GP) addresses and care home registered addresses. Performance was evaluated in a diagnostic test study comparing NLP prediction to independent, gold-standard manual identification of care home addresses.<br>Results: The analysis used population data for 771,588 uniquely written addresses for 819,911 people in two NHS Scotland health board regions. Care home resident identification by NLP methods overall was better in Fife than in Tayside, and better in the over-65s than in the whole population. Methods with the best performance were Correlation (sensitivity 91.2%, PPV 92.0%) for Fife data and Cosine (sensitivity 90.4%, PPV 93.7%) for Tayside. For people aged ≥65 years, the best methods were Jensen-Shannon: (sensitivity 91.5%, PPV 98.7%) for Fife and City Block (sensitivity 94.4%, PPV 98.3%) for Tayside. These results show the feasibility of applying NLP methods to real data concluding that computing address similarities outperforms previous works.<br>Conclusions: Address-matching techniques using NLP methods can determine with reasonable accuracy if individuals live in a care home based on their GP-registered addresses. The performance of the system exceeds previously reported results such as Postcode matching, Markov score or Phonics score.
**Order of Authors:**	Víctor Suárez-Paniagua, Ph.D.<br>Arlene Casey<br>Charis A. Marwick<br>Jennifer K. Burton<br>Helen Callaby<br>Isobel Guthrie<br>Bruce Guthrie<br>Beatrice Alex
**Additional Information:**	
**Question**	**Response**
**Financial Disclosure**<br><br>Enter a financial disclosure statement that	Yes

describes the sources of funding for the work included in this submission. Review the submission guidelines for detailed requirements. View published research articles from *PLOS ONE* for specific examples.<br><br>This statement is required for submission and **will appear in the published article** if the submission is accepted. Please make sure it is accurate.<br><br>**Funded studies**<br>Enter a statement with the following details:<br>• Initials of the authors who received each award<br>• Grant numbers awarded to each author<br>• The full name of each funder<br>• URL of each funder website<br>• Did the sponsors or funders play any role in the study design, data collection and analysis, decision to publish, or preparation of the manuscript?<br><br>Did you receive funding for this work?	
Please add funding details.<br>as follow-up to "**Financial Disclosure**<br><br>Enter a financial disclosure statement that describes the sources of funding for the work included in this submission. Review the submission guidelines for detailed requirements. View published research articles from *PLOS ONE* for specific examples.<br><br>This statement is required for submission and **will appear in the published article** if the submission is accepted. Please make sure it is accurate.	This work was funded by Legal and General PLC (a research grant to establish the independent Advanced Care Research Centre at the University of Edinburgh). The funder had no role in the conduct of the study, interpretation, or the decision to submit for publication. The views expressed are those of the authors and not necessarily those of Legal and General PLC. The work was additionally supported by the HDR UK National Text Analytics Implementation Project, Wellcome Institutional Translation Partnership Awards (PIII029).

**Funded studies**<br>Enter a statement with the following details:<br>• Initials of the authors who received each award<br>• Grant numbers awarded to each author<br>• The full name of each funder<br>• URL of each funder website<br>• Did the sponsors or funders play any role in the study design, data collection and analysis, decision to publish, or preparation of the manuscript?<br><br>Did you receive funding for this work?"	
Please select the country of your main research funder (please select carefully as in some cases this is used in fee calculation).<br>as follow-up to "**Financial Disclosure**<br><br>Enter a financial disclosure statement that describes the sources of funding for the work included in this submission. Review the submission guidelines for detailed requirements. View published research articles from *PLOS ONE* for specific examples.<br><br>This statement is required for submission and **will appear in the published article** if the submission is accepted. Please make sure it is accurate.<br><br>**Funded studies**<br>Enter a statement with the following details:<br>• Initials of the authors who received each award<br>• Grant numbers awarded to each author<br>• The full name of each funder<br>• URL of each funder website<br>• Did the sponsors or funders play any role in the study design, data collection and analysis, decision to publish, or preparation of the manuscript?<br><br>Did you receive funding for this work?"	UNITED KINGDOM - GB
**Competing Interests**	The authors have declared that no competing interests exist.

Use the instructions below to enter a competing interest statement for this submission. On behalf of all authors, disclose any competing interests that could be perceived to bias this work—acknowledging all financial support and any other relevant financial or non-financial competing interests.<br><br>This statement is required for submission and **will appear in the published article** if the submission is accepted. Please make sure it is accurate and that any funding sources listed in your Funding Information later in the submission form are also declared in your Financial Disclosure statement.<br><br>View published research articles from *PLOS ONE* for specific examples.<br><br>**NO authors have competing interests**<br><br>Enter: *The authors have declared that no competing interests exist.*<br><br>**Authors with competing interests**<br><br>Enter competing interest details beginning with this statement:<br><br>*I have read the journal's policy and the authors of this manuscript have the following competing interests: [insert competing interests here]*<br><br>* typeset	
**Ethics Statement**<br><br>Enter an ethics statement for this submission. This statement is required if the study involved:<br><br>• Human participants<br>• Human specimens or tissue<br>• Vertebrate animals or cephalopods<br>• Vertebrate embryos or tissues	N/A

• Field research<br><br>Write "N/A" if the submission does not require an ethics statement.<br><br>General guidance is provided below. Consult the submission guidelines for detailed instructions. **Make sure that all information entered here is included in the Methods section of the manuscript.**	

**Format for specific study types**<br><br>**Human Subject Research (involving human participants and/or tissue)**<br>• Give the name of the institutional review board or ethics committee that approved the study<br>• Include the approval number and/or a statement indicating approval of this research<br>• Indicate the form of consent obtained (written/oral) or the reason that consent was not obtained (e.g. the data were analyzed anonymously)<br><br>**Animal Research (involving vertebrate animals, embryos or tissues)**<br>• Provide the name of the Institutional Animal Care and Use Committee (IACUC) or other relevant ethics board that reviewed the study protocol, and indicate whether they approved this research or granted a formal waiver of ethical approval<br>• Include an approval number if one was obtained<br>• If the study involved *non-human primates*, add *additional details* about animal welfare and steps taken to ameliorate suffering<br>• If anesthesia, euthanasia, or any kind of animal sacrifice is part of the study, include briefly which substances and/or methods were applied<br><br>**Field Research**<br><br>Include the following details if this study involves the collection of plant, animal, or other materials from a natural setting:<br>• Field permit number<br>• Name of the institution or relevant body that granted permission	
**Data Availability**<br><br>Authors are required to make all data underlying the findings described fully available, without restriction, and from the time of publication. PLOS allows rare exceptions to address legal and ethical concerns. See the PLOS Data Policy and FAQ for detailed information.	No - some restrictions will apply

A Data Availability Statement describing where the data can be found is required at submission. Your answers to this question constitute the Data Availability Statement and **will be published in the article**, if accepted.<br><br>**Important:** Stating 'data available on request from the author' is not sufficient. If your data are only available upon request, select 'No' for the first question and explain your exceptional situation in the text box.<br><br>Do the authors confirm that all data underlying the findings described in their manuscript are fully available without restriction?	
**Describe where the data may be found in full sentences. If you are copying our sample text, replace any instances of XXX with the appropriate details.**<br><br>• If the data are **held or will be held in a public repository**, include URLs, accession numbers or DOIs. If this information will only be available after acceptance, indicate this by ticking the box below. For example: *All XXX files are available from the XXX database (accession number(s) XXX, XXX.*).<br>• If the data are all contained **within the manuscript and/or Supporting Information files**, enter the following: *All relevant data are within the manuscript and its Supporting Information files.*<br>• If neither of these applies but you are able to provide **details of access elsewhere**, with or without limitations, please do so. For example:<br><br>*Data cannot be shared publicly because of [XXX]. Data are available from the XXX Institutional Data Access / Ethics Committee (contact via XXX) for researchers who meet the criteria for access to confidential data.*<br><br>*The data underlying the results presented in the study are available from (include the name of the third party*	The datasets presented in this article are not readily available because they can only be accessed by submitting an application to the information governance body: the Health Informatics Centre of the University of Dundee. Requests to access the datasets should be directed to b.alex@ed.ac.uk.

and contact information or URL).<br>• This text is appropriate if the data are owned by a third party and authors do not have permission to share the data.<br><br>* typeset	
Additional data availability information:	

Manuscript

Click here to access/download;Manuscript;CareHomeAddress PLoS.docx

Care home resident identification: A comparison of address matching methods with Natural Language Processing

Víctor Suárez-Paniagua[1,2,*], Arlene Casey[1], Charis A. Marwick[3], Jennifer K. Burton[6], Helen Callaby[7], Isobel Guthrie[8], Bruce Guthrie[1], Beatrice Alex[1,4,5]

[1]Advanced Care Research Centre, Usher Institute of Population Health Sciences and Informatics, University of Edinburgh, Biocube 1, NINE Edinburgh BioQuarter, EH16 4UX, Edinburgh, United Kingdom, [2]Health Data Research UK, HDR UK, London, United Kingdom, [3]Population Health & Genomics Division, School of Medicine, University of Dundee, Ninewells Campus, Dundee DD1 9SY, United Kingdom, [4]School of Literatures, Languages and Cultures (LLC), University of Edinburgh, 50 George Square, Newington, EH8 9LH, Edinburgh, United Kingdom, [5]Edinburgh Futures Institute, University of Edinburgh, 1 Crichton Street, EH8 9AB, Edinburgh, United Kingdom, [6]School of Cardiovascular and Metabolic Health, University of Glasgow, New Lister Building, Glasgow Royal Infirmary, 10 Alexandra Parade, G31 2ER, [7]NHS Tayside, and [8]University of St Andrews School of Biology, Biomedical Sciences Research Complex, St Andrews, KY16 9ST

*Corresponding author. vsuarez@ed.ac.uk

Abstract

Background: Care home residents are a highly vulnerable group, but identifying care home residents in routine data is challenging. This study aimed to develop and validate Natural Language Processing (NLP) methods to identify care home residents from primary care address records.

Methods: The proposed system applies an NLP sequential filtering and preprocessing of text, then the calculation of similarity scores between general practice (GP) addresses and care home registered addresses. Performance was evaluated in a diagnostic test study comparing NLP prediction to independent, gold-standard manual identification of care home addresses.

Results: The analysis used population data for 771,588 uniquely written addresses for 819,911 people in two NHS Scotland health board regions. Care home resident identification by NLP methods overall was better in Fife than in Tayside, and better in the over-65s than in the whole population. Methods with the best performance were **Correlation** (sensitivity 91.2%, PPV 92.0%) for Fife data and **Cosine** (sensitivity 90.4%, PPV 93.7%) for Tayside. For people aged ≥65 years, the best methods were **Jensen-Shannon**: (sensitivity 91.5%, PPV 98.7%) for Fife and **City Block** (sensitivity 94.4%, PPV 98.3%) for Tayside. These results show the feasibility of applying NLP methods to real data concluding that computing address similarities outperforms previous works.

Conclusions: Address-matching techniques using NLP methods can determine with reasonable accuracy if individuals live in a care home based on their GP-registered addresses. The performance of the system exceeds previously reported results such as Postcode matching, Markov score or Phonics score.

Keywords: Care Home, Address Matching, Natural Language Processing, Similarity Measure, Older People

Introduction

Linked routine data is an increasingly powerful tool to understand health and social care needs, patterns of care and outcomes. However, key groups of vulnerable people are relatively invisible in routine data, including residents of care homes [1, 2]. This was brutally exposed by the COVID-19 pandemic, where the inability to identify care home residents in routine data seriously constrained pandemic response in a population in which a large proportion of COVID-19 deaths occurred [3, 4, 5]. Existing methods for identifying care home residents in routine data typically involve methods which are known to be fallible (although this is rarely quantified) or require considerable amounts of manual matching of addresses [6, 7, 8]. Beyond COVID-19, care home residents are a highly vulnerable population with high use of planned and unscheduled healthcare, but the inability to identify them accurately in routine data is a barrier to understanding their needs and improving their care [2].

Identifying care home residents in routine data sources is a challenging task [9, 10]. Currently, in the UK identification typically relies on coding in the primary healthcare record (which is very incomplete) or various forms of address matching between the general practitioner (GP) recorded address and the addresses of care homes registered with the regulator.

Natural Language Processing (NLP), a subfield of Artificial Intelligence (AI) has the potential to classify care home residents from their addresses automatically using state-of-the-art Computer Science algorithms. Concretely, NLP methods applied to address matching can be used to extract the relevant information of addresses to check whether a patient belongs to any official care home service.

Previous work using this approach include Sherlaw-Johnson et al. [11], who examined hospital admission from postcodes, Burton et al. [4] who explored five methods to classify GP-recorded Community Health Index (CHI) addresses as being care home addresses, Schultze et al. [13] who compared three methods of identifying care home residents in primary healthcare data, and Santos et al. [9] who proposed using the Ordnance Survey AddressBase Premium database [14] for care home address matching from people aged 65 years old or over registered by the Care Quality Commission [15] in England.

This study aims to develop NLP methods for identifying care home residents in primary healthcare data and validate their performance in a realistic scenario using a gold standard selected from real population data.

Methods

Overall design

The overall design is a diagnostic test study applying and optimizing address-matching methods with NLP to identify care home residents in a training dataset, with

a comparison with previous methods [12] in an independent validation dataset. Figure 1 shows the complete pipeline of the proposed method.

Figure 1: Address matching system pipeline.

Datasets

The University of Dundee Health Informatics Centre (HIC) provided a list of unique addresses recorded in the NHS Scotland Master CHI registers for residents of NHS Tayside and NHS Fife up to 30th April 2020. CHI addresses are those stored nationally based on information submitted at the time of registration with a general practitioner (GP) practice for primary health care provision. Patient identifiers were removed from the dataset. The number of residents living at each unique address on 30th April 2020 was also provided, and whether at least one person aged 65 years or over currently lived at that address (a unique address means 'as recorded at GP registration', where the same actual address may have different unique forms, e.g. 10 Glebe Street, Ten Glebe Street, 10 Glebe St. etc.). These datasets were randomly and proportionally split into 70% and 30% for training and validation, respectively (Table 1). Care home addresses were obtained from publicly available data from the Care Inspectorate, the Scottish care regulator, who records care home registered addresses [16].

Table 1: Number of historical and current unique addresses in the Community Health Index (CHI) and number of people living at a CHI address on 30th April 2020.

	Unique historical or current addresses in CHI[a]	No. (%) of care home historical or current addresses in CHI[b]	No. of people living at a CHI address on 30/4/20[c]	No. (%) of care home residents on 30/4/20[c]
**All ages**				
**Fife**	**363,091**	4,851 (1.3%)	**391,081**	2,829 (0.7%)
Training set (70%)	254,164	3,396 (1.3%)	273,207	1,940 (0.7%)
Validation set (30%)	108,927	1,455 (1.3%)	117,874	889 (0.8%)
**Tayside**	**408,497**	5,244 (1.3%)	**428,830**	3,331 (0.8%)
Training set (70%)	285,949	3,671 (1.3%)	300,448	2,492 (0.8%)
Validation set (30%)	122,548	1,573 (1.3%)	128,382	839 (0.6%)
**Aged ≥65 years**				
**Fife**	**60,748**	608 (1.0%)	**91,575**	2,685 (2.9%)
Training set (70%)	42,524	426 (1.0%)	64,173	1,919 (3.0%)
Validation set (30%)	18,224	182 (1.0%)	27,402	766 (2.8%)
**Tayside**	**69,475**	413 (0.6%)	**106,844**	3,224 (3.0%)
Training set (70%)	48,634	290 (0.6%)	74,732	2,378 (3.2%)
Validation set (30%)	20,841	123 (0.6%)	32,112	846 (2.6%)

a. Includes both current and historical addresses (true addresses do not change much over time, but unique addresses recorded in CHI are free-text so the same true address can have multiple different versions in CHI over time).

b. CHI addresses manually identified as care home addresses (gold-standard). This includes historical addresses where no one is resident on 30/4/20

c. The number of people living at a CHI address on 30/4/20, or the number of people living at a gold-standard care home identified care home address on 30/4/20. Care home addresses are more variable in how they are written than other addresses, and there are large numbers of small care homes for younger people, so for all ages, there are more historical or current care home addresses than current residents.

Reference standard

Five assessors undertook manual allocation of all CHI addresses to determine if the address was a care home address or not, referring to the Care Inspectorate list of services. This manually allocated list was used as the gold standard for this study.

Data filtering and pre-processing

CHI addresses recorded at GP registration are free-text fields without any required format specification (e.g., the postcode is not required to be in the correct order or appear on its own on a line, and the complete address may be written on one line). Before calculating similarity measures, the datasets were prepared and cleaned using a filtering process and a pre-processing step.

The filtering process had three steps (Figure 1). First, the CHI address was classified as a care home address if the first line contained any phrase related to a care home name such as "care home", "nursing home" or "residential home". Second, the CHI address was classified as *not* being a care home address if the CHI and care home postcodes did not completely exactly match *or* the care inspectorate registered address was for a different kind of care services *or* the CHI address did not include the name of a town contained in any of the care home addresses (Appendix A). Third, the remaining unclassified addresses were passed to the pre-processing step.

In the preprocessing step, CHI and care home registered addresses were cleaned as follows: address lines containing commas were split into separated lines, and regular expressions were used to extract numbers together with adjacent letters (excluding spaces and non-alphanumeric characters such as '/'), and all strings were converted to lower case. For example, following these processes, the address line '*89 Bay Street, Newtown-on-Bay*' would be divided into two new address lines and strings cleaned as '[*bay street*, *newtown on bay*]' and a list of numbers '[*89*]'. At the end of this step, the processed address lines are stored as a string or transformed into a vector representation (Appendix B).

Address matching methods

Since both CHI and care home registered addresses are free-text fields, exact address matching is not feasible. Three methods previously applied to these data were applied as baseline comparators: Postcode matching, a Markov method, and a Phonics method [12].

Postcode matching considers a given CHI address as a care home address if its postcode is identical to any postcode in the official care home list. Identifying care home residency in this way is not very accurate overall because the same postcode is often given to different properties. However, this method was validated in previous studies, so we also report it alongside new approaches.

The Markov and Phonics methods create a similarity score where the CHI address is defined as that of a care home if the score is above a defined threshold. In this study,

we applied the thresholds proposed in the previous study and (to enable a fair comparison with new methods) optimized thresholds in the training set.

We then applied 11 additional methods for similarity-matching addresses and optimized them using the training datasets to choose the correct cutoff. All 11 methods calculate a similarity score between addresses (Appendix E): four using approximate (or fuzzy) string matching to calculate edit distance (Appendix C), and seven using vector space models (Appendix D).

Evaluation

The performance of all baseline and new methods was evaluated in terms of positive predictive value (PPV), sensitivity, negative predictive value (NPV), specificity, and the F1 measure. F1 is the harmonic mean of PPV and sensitivity (Appendix F). Since being resident in a care home is a relatively rare event within the older adult population, the key performance characteristics desired are high PPV (almost all of those identified as care home residents truly are residents) and high sensitivity (almost all actual care home residents are identified as residents).

The main objective of this research is to determine which unique addresses are care homes (address level). From a more practical perspective, we can recognize which patients with the resident status can be recognized by knowing the number of people living in each address (patient level). For this reason, the evaluation was carried out at the address level (matching unique addresses) and at the patient level (matching the number of people in each address) using the entire historical dataset on 30th April 2020.

Results

A total of 771,588 unique CHI current and historical address records for 819,911 people were analysed, of which 408,497 unique addresses for 428,830 were in NHS Tayside and 363,091 unique addresses for 391,081 people were in NHS Fife. Table 2 shows the performance of the methods for the validation set at the address and patient levels.

Table 2: Positive predictive value, sensitivity and their harmonic mean F1 for each method of identifying care home *addresses* in the CHI address dataset, compared to gold-standard manual identification.

Population	Method	PPV	Sensitivity	F1
Fife all addresses	Markov score[a]	78.0%	77.3%	77.6%
	City block	79.5%	72.1%	75.6%
	**Bray-Curtis**	83.7%	77.0%	**80.2%**
	Cosine	85.4%	70.9%	77.5%
	Jensen-Shannon	76.7%	76.0%	76.3%
Tayside all addresses	Markov score[a]	66.5%	57.2%	61.5%
	City block	69.6%	64.6%	67.0%
	Bray-Curtis	66.8%	70.4%	68.6%
	**Cosine**	77.1%	64.0%	**69.9%**
	Jensen-Shannon	68.8%	64.1%	66.4%
Fife addresses with ≥1 person aged ≥65 years	Markov score[a]	90.8%	76.4%	83.0%
	City block	96.7%	80.2%	87.7%
	Bray-Curtis	90.5%	84.1%	87.2%
	Cosine	92.6%	82.4%	87.2%
	**Jensen-Shannon**	96.2%	83.0%	**89.1%**
Tayside addresses with ≥1 person aged ≥65 years	Markov score[a]	70.0%	79.7%	74.5%
	**City block**	94.5%	83.7%	**88.8%**
	Bray-Curtis	92.5%	80.5%	86.1%
	Cosine	95.3%	82.1%	88.2%
	Jensen-Shannon	95.2%	81.3%	87.7%

a. Markov score using the same methods as Burton e al [12] but cut-offs fine-tuned for this dataset (allocated as care home when Markov score >47 in Fife all, >62 in Fife ≥65, >37 in Tayside all, >45 in Tayside ≥65).

In the evaluation, we carried out different ablation studies and parameter tuning to find the best configuration for the care home address matching system pipeline such as: comparing the filtering processes (Appendix A) and the representation of the addresses with the n-gram hyperparameter (Appendix B). In addition, we optimized and compared the different edit distance approaches (Appendix C) and the vector space models (Appendix D) to find the best performance for each population.

We replicated the three previously examined methods [12] finding that the Markov score method performed better than the Phonics score method or the postcode matching, and we compared them with the 11 newly applied methods (Appendix E). Therefore, we used the Markov score (with cut-offs from the original study, and cut-offs optimized for this dataset) to compare the four best methods in Tables 2 and 3. General patterns were that: performance was better for Fife data than Tayside data;

performance was better in the over-65 population compared to all ages; and the newer methods almost all outperformed the Markov score (fined tuned for this dataset to allow a fair comparison).

The methods which overall performed best at identifying if a CHI address was a care home address (compared to gold-standard manual allocation) for all ages were **Bray-Curtis** (F1 80.2%, sensitivity 77.0%, PPV 83.7%) for the Fife data and **Cosine** (F1 69.3%, sensitivity 64.0%, PPV: 77.0%) for the Tayside data (Table 2). Performance was better for people aged ≥65 years, whereas for addresses where at least one person aged ≥65 years lived, the methods with the best performance were **Jensen-Shannon** (F1 94.5%, sensitivity 83.0%, PPV 96.2%) for Fife and **City Block** (F1 88.8%, sensitivity 83.7%, PPV 94.5%) for Tayside. However, differences between methods were relatively small (for example, in the Tayside over-65 population, the worse performing new method was Bray Curtis [F1 86.1%, sensitivity 92.5%, PPV 86.1%]).

The methods with the overall best performance for identifying whether an individual was resident in a care home for all ages were **Correlation** (F1 91.2%, sensitivity 91.2%, PPV 92.0%) for Fife data and **Cosine** (F1 92.0%, sensitivity 90.4%, PPV 93.7%) for Tayside (Table 3). Performance was higher for people aged ≥65 years, with the best methods being **Jensen-Shannon**: (F1 95.0%, sensitivity 91.5%, PPV 98.7%) for Fife and **City Block** (F1 96.3%, sensitivity 94.4%, PPV 98.3%) for Tayside. Again, differences between methods were relatively small (for example, in the Fife over-65 population, the worse performing new method was City Block (F1 94.4%, sensitivity 99.0%, PPV 90.2%).

Table 3: Positive predictive value, sensitivity and their harmonic mean F1 for each method of identifying care home *residents* in the CHI address dataset, compared to gold-standard manual identification.

Population	Method	PPV	Sensitivity	F1
Fife all people	Markov score[a]	77.0%	88.1%	82.2%
	City block	82.1%	88.8%	85.3%
	Cosine	92.6%	88.5%	90.5%
	Jensen-Shannon	77.0%	90.0%	83.0%
	**Correlation**	92.0%	90.2%	**91.1%**
Tayside all people	Markov score[a]	71.9%	89.6%	79.8%
	City block	72.1%	92.9%	81.2%
	**Cosine**	93.7%	90.4%	**92.0%**
	Jensen-Shannon	70.1%	92.3%	79.7%
	Correlation	86.0%	91.5%	88.7%
Fife people aged ≥65 years	Markov score[a]	96.7%	84.2%	90.0%
	City block	99.0%	90.2%	94.4%
	Cosine	97.8%	91.6%	94.6%
	**Jensen-Shannon**	98.7%	91.5%	**95.0%**
	Correlation	98.3%	91.8%	94.9%
Tayside people ≥65 years	Markov score[a]	91.6%	86.4%	88.9%
	**City block**	98.3%	94.4%	**96.3%**
	Cosine	98.4%	94.2%	96.3%
	Jensen-Shannon	98.4%	94.1%	96.2%
	Correlation	98.4%	94.2%	96.3%

a. Markov score using the same methods as Burton e al [12] but cut-offs fine-tuned for this dataset (allocated as care home when Markov score >47 in Fife all, >62 in Fife ≥65, >37 in Tayside all, >45 in Tayside ≥65).

Discussion

Summary of findings

We validated several address-matching methods to identify care home residents using a large real sample covering the entire population of two health boards in Scotland, NHS Fife and NHS Tayside. Specifically, we developed a pipeline that scores the similarity of a patient address to any of the addresses in a list of Care Inspectorate registered care home services. To do so, we first applied a rule-based filtering process to the GP-recorded addresses in the CHI dataset to deal with special cases, and a preprocessing step using regular expressions to clean the remaining address lines and transform them into a string or vector. Finally, each address was then compared to the

official care home list using different similarity-matching methods and classified as a care home address if the computed score was below a pre-trained threshold. We compared three baseline methods proposed in previous work and variations of two types of distance approaches: the edit distance, which quantifies the number of character changes required to transform one address into another, and the vector space model, which transforms each address into a numeric vector. In the experiments, we observed that in general vector space models outperform edit distance ones.

The new methods had higher performance than the best of the baseline methods (the Markov score approach [12]). However, performance varied depending on the population examined. Identification of care home addresses and residents was systematically better in Fife than in Tayside and was systematically better for older people (aged 65 years and over) compared to the whole population. Performance was also better at identifying care home residents than identifying care home addresses reflecting that the addresses with a low number of care home residents are harder to identify.

Strengths and limitations

This study demonstrates that NLP methods can be effectively applied to address matching to determine if a patient's address is that of a care home. We used a large sample size from two regions of Scotland with the mixed city, town, and rural addresses. We tested our methods against a gold-standard researcher-produced dataset. While this study was developed and validated using data extracted on 30th April 2020, the presented methods would work on future samples, if the official care home list is up to date to guarantee successful matching between a new CHI address and a care home on service. A key limitation is that the variation in performance between the two regions means that performance may also vary when applied in other parts of the UK, and ideally anyone implementing these methods will validate locally.

Comparison with other literature

Sherlaw-Johnson et al. examined hospital admission from postcodes known to contain a single care home and observed an unusually high rate of admission from these postcodes [11], but the analysis included people living in other dwellings with the same postcode and excluded postcodes with more than one care home. Burton et al. explored five methods to classify GP-recorded addresses as being care homes with a gold standard sample of 20,000 addresses which had been manually allocated as belonging to a care home or not [12]. Although some of the evaluated methods had excellent performance in identifying care homes in terms of PPV or sensitivity individually, none of them obtained good results in both metrics simultaneously. Schultze et al. compared three methods of identifying care home residents in primary healthcare data (coded as a resident; using address matching; and using the primary care record 'household' identifier to identify households with multiple older residents although this will include sheltered housing residents), with coded data identifying

the fewest and household identifiers the most potential residents, with only partial overlap [13]. However, their study had no gold-standard method for identifying residents and therefore no means of evaluating the accuracy of allocation.

Recently, Santos et al. proposed using the Ordnance Survey AddressBase Premium database [14] for care home address matching. AddressBase Premium contains all addresses in the UK and assigns a Unique Property Reference Number (UPRN) to each address. The study collected data from people aged 65 years old or over registered by the Care Quality Commission [15] in England with the same postcode of a care home as potential care home residents. Then, the UPRN matching was validated using a manual address-matched gold standard and obtained 78.0% sensitivity and 99.4% PPV. These results are promising, but the study only included 80 English care home addresses out of 15,019 (0.5%), so the feasibility of real population data is unclear. Moreover, Zhang et al. [17] proposed a machine-learning based model, called FLAP, to link UPRN from free-text addreses that showed a reasonable error tolerance in writing and obtained a 99.2% of Accuracy in the experiments.

While previous works had focused on improving the PPV or sensitivity individually, the present study aimed to obtain the best model that can perform well in both scenarios optimizing the F1 metrics which is its harmonic mean. Compared to the Postcode Matching baseline also reported on in [12], the performance of the best configurations improves from 77.64% to 80.21% F1 using **Bray-Curtis** in Fife at address level (from 82.16% to 91.08% F1 using **Correlation** at patient level) and from 61.5% to 69.93% F1 using **Cosine** in Tayside at address level (from 79.79% to 91.99% F1 using **Cosine** at patient level). For the population aged 65 or over, F1 increased from 82.99% to 89.09% using **Jensen-Shannon** in Fife at the address level (from 90.02% to 94.99% using **Jensen-Shannon** at the patient level) and from 74.52% to 88.79% using **City Block** in Tayside (from 88.93% to 96.32% using **City Block** at patient level).

Implications of the study

An integrated health and social care data system would enable care homes to share information about residents moving in and out of their services, which can be linked to other data to build up a picture of their care journey. Address matching of GP-recorded addresses to care home registered addresses is a reasonably effective way of identifying (permanent) care home residents and has the potential to significantly improve our understanding of the needs, service use and outcomes of this vulnerable population. Potential ways of further improving matching would include the use of novel machine learning methods to perform the similarity matching between addresses such as, ensemble methods which use all the different matching scores to classify the resident condition or using state-of-the-art AI methods such as the Siamese Neural Network that can predict the similarity between two addresses [18]. As Santos et al proposed, it may also be more efficient to map both GP-recorded addresses and care home-registered addresses to the Unique Property Reference Number (UPRN) in the Ordnance Survey AddressBase dataset and use UPRN as the linking field. In both cases, the task would be made simpler if GP registration used an

online look-up for addresses to ensure that they were recorded in a consistent way (in the way that online shopping does). Alternatively, rather than identifying care home addresses using an inevitably less-than-perfect method, a more systematic recording of who is a care home resident in routine data would be optimal, which will require care homes to securely share these data with public agencies [2].

Conclusion

The inability to identify care home residents in routine data makes this highly vulnerable population relatively invisible in research and policy. Address matching using NLP methods can identify care home residents with high performance using realistic population data, and in the absence of systematic recording in routine data, can underpin research and understanding for planning and policy.

Author contributions statement

VSP conceived the research, planned the study, designed the system, performed the implementation, conducted the experiments, and analyzed the results. VSP wrote and reviewed the manuscript with support from AC, HZ, CAM, JKB, HC, IG, BG, HW and BA. CAM, JKB, HC, IG, BG annotated the data.

Acknowledgements

The authors would like to thank members of the Clinical Natural Language Processing Research Group and KnowLab at the University of Edinburgh and University College London for their valuable discussion and comments. We are grateful to the University of Dundee Health Informatics Centre for the data provision and technical support.

Funding

This work was funded by Legal and General PLC (a research grant to establish the independent Advanced Care Research Centre at the University of Edinburgh). The funder had no role in the conduct of the study, interpretation, or the decision to submit for publication. The views expressed are those of the authors and not necessarily those of Legal and General PLC. The work was additionally supported by the HDR UK National Text Analytics Implementation Project, Wellcome Institutional Translation Partnership Awards (PIII029).

Conflicts of interest

None.

References

1. Burton JK, Guthrie B. Identifying who lives in a care home—a challenge to be conquered. Age and Ageing 2018;47:322-3

2. Burton J, Goodman C, Guthrie B, Gordon A, Hanratty B, Quinn T. Closing the UK care home data gap – methodological challenges and solutions: Closing the UK care home data gap. International Journal of Population Data Science 2020;5:doi: 10.23889/ijpds.v5i4.1.

3. Hanratty, B., Burton, J., Goodman, C., Gordon, A., & Spilsbury, K. (2020). Covid-19 and lack of linked datasets for care homes. BMJ, 369.

4. Burton JK, Bayne G, Evans C, et al. Evolution and effects of COVID-19 outbreaks in care homes: a population analysis in 189 care homes in one geographical region of the UK. The Lancet Healthy Longevity 2020;1:e21-e31.

5. Burton JK, McMinn M, Vaughan JE, Fleuriot J, Guthrie B. Care-home outbreaks of COVID-19 in Scotland March to May 2020: National linked data cohort analysis. Age and Ageing 2021;50:1482-92.

6. Public Health Scotland, University of Edinburgh, University of Glasgow. Discharges from NHSScotland hospitals to care homes between 1 March and 31 May 2020: A Management Information Statistics publication for Scotland. Edinburgh: Public Health Scotland; 2021.

7. Hollinghurst J, Lyons J, Fry R, et al. The impact of COVID-19 on adjusted mortality risk in care homes for older adults in Wales, UK: a retrospective population-based cohort study for mortality in 2016–2020. Age and Ageing 2020.

8. Emmerson C, Adamson JP, Turner D, et al. Risk factors for outbreaks of COVID-19 in care homes following hospital discharge: A national cohort analysis. Influenza and Other Respiratory Viruses 2021;15:371-8

9. Filipe Oliveira dos Santos, Stefano Conti, and Arne Wolters. A novel method for identifying care home residents in England: A validation study. International Journal of Population Data Science, 5(4), Sep. 2021.

10. Danielle Collingridge Moore and Barbara Hanratty. Out of sight, out of mind? a review of data available on the health of care home residents in longitudinal and nationally representative cross-sectional studies in the UK and Ireland. Age and Ageing, 42(6): 798–803, 2013.

11. Chris Sherlaw-Johnson, Paul Smith, and Martin Bardsley. Continuous monitoring of emergency admissions of older care home residents to hospital. Age and Ageing, 45(1): 71–77, 11 2015.

12. Jennifer K Burton, Charis A Marwick, James Galloway, Christopher Hall, Thomas Nind, Emma L Reynish, and Bruce Guthrie. Identifying care-home residents in routine

healthcare datasets: a diagnostic test accuracy study of five methods. Age and Ageing, 48(1): 114–121, 07 2018.

13. Schultze A, Bates C, Cockburn J, et al. Identifying Care Home Residents in Electronic Health Records - An OpenSAFELY Short Data Report [version 1; peer review: 1 approved]. Wellcome Open Research 2021;6.

14. Ordnance Survey – AddressBase Premium. https://www.ordnancesurvey.co.uk/business-government/products/addressbase-premium.

15. Care Quality Commission (CQC). https://www.cqc.org.uk.

16. Care inspectorate. https://www.careinspectorate.com.

17. Zhang, Huayu and Casey, Arlene and Guellil, Imane and Suárez-Paniagua, Víctor and MacRae, Clare and Marwick, Charis and Wu, Honghan and Guthrie, Bruce and Alex, Beatrice. FLAP: a framework for linking free-text addresses to the Ordnance Survey Unique Property Reference Number database. Frontiers in Digital Health 2023, 5. doi: 10.3389/fdgth.2023.1186208.

18. Reimers, Nils and Gurevych, Iryna. Sentence-BERT: Sentence Embeddings using Siamese BERT-Networks. Proceedings of the 2019 Conference on Empirical Methods in Natural Language Processing. Association for Computational Linguistics11-2019.

19. GOV.UK – Bulk Data Transfer. https://assets.publishing.service.gov.uk/government/uploads/system/uploads/attachment_data/file/488478/Bulk_Data_Transfer_-_additional_validation_valid_from_12_November_2015.pdf.

20. V. I. Levenshtein. Binary Codes Capable of Correcting Deletions, Insertions and Reversals. Soviet Physics Doklady, 10: 707, February 1966.

21. Fred J. Damerau. A technique for computer detection and correction of spelling errors. Commun. ACM, 7(3): 171–176, March 1964.

22. Matthew A. Jaro. Advances in record-linkage methodology as applied to matching the 1985 census of Tampa, Florida. Journal of the American Statistical Association, 84(406): 414–420, 1989.

23. William E. Winkler. String comparator metrics and enhanced decision rules in the fellegi-sunter model of record linkage. In Proceedings of the Section on Survey Research, pages 354–359, 1990.

24. Grzegorz Kondrak. N-gram similarity and distance. In Mariano Consens and Gonzalo Navarro, editors, String Processing and Information Retrieval, pages 115–126, Berlin, Heidelberg, 2005. Springer Berlin Heidelberg.

25. Karl Pearson. Note on Regression and Inheritance in the Case of Two Parents. Proceedings of the Royal Society of London Series I, 58:240–242, January 1895.

26. S. Kullback and R. A. Leibler. On information and sufficiency. Ann. Math. Statist., 22(1): 79–86, 03 1951.

27. G. Lance and W. T. Williams. A general theory of classificatory sorting strategies: 1. hierarchical systems. Comput. J.,9:373–380, 1967.

28. J. Roger Bray and J. T. Curtis. An ordination of the upland forest communities of southern Wisconsin. Ecological Monographs,27(4):325–349, 1957

Supplementary material

Appendix A: Filtering selection

The filtering process determines conditions to be met before processing using simple rules and basic comparisons developed during initial data exploration. For example, lack of postcode match was found to be a good discarding indicator with a low number of false negatives. Filtering involves the application of four different conditions:

1. **CHname**: whether the patient's first address line contains a string related to a care home name such as "care home", "nursing home", or "residential home". If so, the similarity is directly assigned to 0, which means this CHI address is labelled as a care home address. Otherwise, the process continues evaluating the following conditions.

2. **PCmatch**: checks if the patient and the care home postcodes are valid using a regular expression extracted from the guidance on bulk data transfer available in the Government information website of the United Kingdom [19] and if they match when both are transformed to lowercase, and spaces are removed.

3. **CHserv**: filters the care home by its type of service using a predefined list of target services ("*Older People*" and "*Physical and Sensory Impairment*" i.e., excluding services for people with learning disabilities, alcohol misuse or mental health).

4. **CHtown**: uses the town in the care home address and tries to find a string match in the different CHI address lines.

The pipeline continues to the next step only if the **PCmatch**, **CHserv**, and **CHtown** conditions are true, otherwise, the similarity is assigned to 1, which means the patient and care home addresses are not considered a match. Tables S1 and S2 present the performance of different combinations of filters using the Damerau-Levenshtein with 1-char-length n-gram to determine the best configuration for address and patient levels, respectively.

Appendix B: Tuning n-gram

The n-gram length is an important hyper-parameter to be tuned because it determines the number of characters (chars) to be compared by the edit distance and to create the vector space model. Thus, it was validated for the string and vector representations using the conditions **PCmatch**, **CHserv**, and **CHname** as filtering processes with the Damerau-Levenshtein and the Cosine distances, respectively. The 2-chars-length n-grams for the edit distance calculation got slightly better results than the 1-char-length, especially in the PPV metrics for the Tayside health board (see Tables S3 and S4 for address and patient levels, respectively). Similarly, the Cosine distance calculated with 1-char-length performs better for the Fife health board while the 2-chars-length achieves greater results in the Tayside addresses and patient level

recognition (see Tables S5 and S6 for address and patient levels, respectively). In this case, the combination of 1 and 2 chars to form the vector representation for each address does not reach the performance of only using 1 or 2 chars. To summarize, according to the results, setting character n-grams to 2 achieves better results in Tayside while 1-grams obtain higher performance in Fife.

Appendix C: Edit distance

Approximate string matching (also known as fuzzy string matching) is a group of methods that calculate the edit distance, which is the minimum number of changes required to transform one string into another. Contrary to exact string matching, these methods give a similarity score being low when the strings are similar. The following are the edit distances used for the experiments:

- **Levenshtein distance** [20] is a string metric that calculates the distance between two string inputs using the operations of insertion, deletion, and substitution.
- **Damerau–Levenshtein distance** [21] is a string metric that includes the transposition of two adjacent characters together with the insertion, deletion, and substitution operations.
- **Jaro distance** [22] considers only the minimum number of character transpositions as the operation to measure the edit distance.
- **Jaro-Winkler distance** [23] is a modification of the previous metric that gives more relevance to the prefixes with a predefined length of the strings.

To normalize the score given by these similarity metrics between 0 and 1 for the experiments, we divide the Levenshtein and Damerau–Levenshtein distances by the maximum length for both inputs. The edit distance computation calculates each operation character by character but, this can be generalized to n characters (n-grams). This generalization outperforms the one-character edit distance because it takes more information about the context [24].

Appendix D: Vector space model

Text documents can be transformed into numeric representations using vector space models. Each dimension in these vectors represents a value of each term in a dictionary. These values are counts of the occurrences for the terms presented in the document, while the remaining terms in the dictionary are 0.

The single characters of the alphabet from a to z and the numbers from 0 to 9 are considered the dictionary of terms for the addresses. In addition, we extended the vocabulary to a higher number of characters (n-grams), thus, we can increase the representation of each address using dictionaries with more than one character and concatenate vocabularies of different sizes to form a vector. For instance, the address

line "89 Bay Street" is separated into *{"8":1, "9":1, " ":2, "t":3, "a":1, "y":1, "s":1, "r":1, "e":2}* using a single-character dictionary, into *{"89":1, "9 ":1, " t":1, "ba":1, "ay":1, "y ":1, " s":1, "st":1, "tr":1, "re":1, "ee":1, "es":1}* using a two-character dictionary, and the concatenation of both representations if single-character and two-character dictionaries are being used together.

Once each address is transformed into vector representations, spatial distance can be computed to measure the similarity between them. These are the distance functions applied to two numeric vectors:

- **City Block (Manhattan)** represents the absolute differences between a pair of vectors u and v, defined as

$$\sum_i |(u_i - v_i)|$$

  and normalized with L_1-norm to be in the range 0,1.

- **Euclidean** represents the root of the square differences between a pair of vectors u and v, defined as

$$\sqrt{\sum_i (u_i - v_i)^2}$$

  and normalized with L_2-norm to be in the range 0,1.

- **Cosine** measures the cosine of the angle between a pair of vectors u and v. Calculated as

$$\frac{\sum_i u_i v_i}{\sqrt{\sum_i u_i^2}\sqrt{\sum_i v_i^2}}$$

- **Correlation** based on the Pearson correlation coefficient [25], measures the covariance of a pair of vectors u and v divided by the root of the multiplication of their square variances. Calculated as

$$\frac{\sum_i (u_i - u^{-})(v_i - v^{-})}{\sqrt{\sum_i (u_i - u^{-})^2}\sqrt{\sum_i (v_i - v^{-})^2}}$$

  being u^{-} and v^{-} the mean of the vectors u and v, respectively. and divided by 2 to be in the range 0,1.

- **Jensen-Shannon** is calculated using the Kullback-Leibler divergences (D) [26] with the probability vectors p and q to their pointwise mean m as

$$\sqrt{\frac{D(p||m) + D(q||m)}{2}}$$

- **Canberra** [27] represents the absolute difference divided by the sum of the vectors u and v, defined as

$$\sum_i \frac{|u_i - v_i|}{|u_i| + |v_i|}$$

- **Bray-Curtis** [28] represents the sum of the fractions between each absolute difference and the absolute additions of the vectors u and v, defined as

$$\frac{\sum_i |u_i - v_i|}{\sum_i |u_i + v_i|}$$

Appendix E: Similarity score computation

Once the address lines and the list of numbers are preprocessed for the patient and care home addresses, Algorithm 1 is applied to calculate the similarity score between both. This step determines the minimum similarity measure for a CHI address line to each line of a care home address using a predefined distance approach. In the case of the lists of numbers for CHI and care home addresses the scores are calculated by exact matching, being 0 if they are the same and 1 if they are different. The minimum similarity measures for each CHI address line and numbers to a care home address are averaged to get a score, which represents the final similarity between them.

*Algorithm 1: Calculate the similarity score 0,1 (being 0 when they are the same address) between a given CHI address (pAddr) and a list of care homes addresses (**chAddrs**).*

```
procedure ADDRESS_SIMILARITY(pAddr, chAddrs)
    for i, pLine in pAddr.lines do
        for j, chLine in chAddr.lines do
            distance_j ← distance(pLine, chLine)
        end for
        line_simil_i ← min_j(distance_j)
    end for
    for i′, pNum in pAddr.numbers do
        for j′, chNum in chAddr.numbers do
            distance_j′ ← not(pNum==chNum)
        end for
        line_simil_i′ ← min_j′(distance_j′)
    end for
    similarity ← ∑_I line_simil_i + ∑_i′ line_simil_i′
    similarity ← similarity/(i+i′)
```

16: **end procedure**

The whole process is repeated over the official care home list. Thus, the final score to find the possible residence status is the minimum similarity measure between a CHI address and all the care home addresses. In the training process, a threshold was applied as a cut-off value to assign the care home resident status to those addresses with a score below the threshold. Figure S1 shows an example of how performance for PPV, sensitivity and F1 measures change when varying the similarity threshold value during the training phase. F1 is included in this research as an NLP standard metric to optimize PPV and sensitivity at the same time. It can be observed that the sensitivity is low at a low threshold because all addresses are recognized as non-residents, but PPV is high because addresses identified as care homes are essentially exact matches. With an increasing threshold, sensitivity increases addresses are matched to those of care homes, but PPV decreases.

Figure S1: Behaviour of system performance measured in PPV (in red), sensitivity (in green) and F1 (in blue) using the training set with an increasing threshold for similarity score (0=exact match, 1=no similarity). The black dot represents the threshold that maximizes the F1 measure.

The final threshold is chosen to maximize a measured value (F1 in the experiments) using the training set. Later, the performance for a specific distance approach is measured using the pre-trained threshold in the validation set. For the experiments, the CHI address dataset was divided into training and validation sets. For each health board (Tayside and Fife) overall and 65 and over populations, data sets are split into 70% for training and 30% for validation of the total sample, respectively. In each set, we ensured the same proportion of positive and negative instances, i.e., CHI addresses which were manually labelled as matching a care home address or not.

Detailed results of the performance in each health board are presented for the baseline methods in Tables S7 and S8, and for the proposed methods in Tables S9 and S10, both using the address level and patient level, respectively.

Appendix F: performance measures

Performance was evaluated using Positive Predictive Value (PPV), Sensitivity, Negative Predictive Value (NPV), Specificity, and an F1 metric, which is the harmonic mean of PPV and Sensitivity, all of which are defined using the numbers of True Positives (TP), True Negatives (TN), False Positives (FP) and False Negatives (FN) of the classifiers compared to gold-standard manual allocation as follows:

$$PPV = \frac{TP}{TP + FP}$$

$$Sensitivity = \frac{TP}{TP + FN}$$

$$NPV = \frac{TN}{TN + FN}$$

$$Specificity = \frac{TN}{TN + FP}$$

$$F1 = 2 \times \frac{PPV \times Sensitivity}{PPV + Sensitivity}$$

Table S1: Results for different filtering approaches at the address level in the validation set to validate the best configuration calculated using the Damerau-Levenshtein distance with 1-char-length n-gram. Bold numbers show the best result for each metric in each health board and population.

Method	Health board and data subset	PPV addresses	Sensitivity addresses	NPV addresses	Specificity addresses	F1 addresses
Damerau–Levenshtein (no filter)	Fife all residents	59.49%	41.58%	99.21%	99.62%	48.95%
	Fife ≥65 years	70.69%	45.05%	99.45%	99.81%	55.03%
	Tayside all residents	51.11%	45.52%	99.29%	99.43%	48.15%
	Tayside ≥65 years	61.22%	48.78%	99.7%	99.82%	54.3%
Damerau–Levenshtein (**PCmatch**)	Fife all residents	81.7%	56.77%	99.42%	99.83%	66.99%
	Fife ≥65 years	81.53%	70.33%	99.7%	99.84%	75.52%
	Tayside all residents	66.28%	53.97%	99.4%	99.64%	59.5%
	Tayside ≥65 years	86.73%	69.11%	99.82%	99.94%	76.92%
Damerau–Levenshtein (**PCmatch**, **CHtown**)	Fife all residents	80.63%	49.21%	99.32%	99.84%	61.12%
	Fife ≥65 years	79.56%	59.89%	99.6%	99.84%	68.34%
	Tayside all residents	66.13%	51.88%	99.38%	99.65%	58.14%
	Tayside ≥65 years	83.65%	70.73%	99.83%	99.92%	76.65%
Damerau–Levenshtein (**PCmatch**, **CHserv**)	Fife all residents	72.78%	65.43%	99.53%	99.67%	68.91%
	Fife ≥65 years	87.94%	68.13%	99.68%	99.91%	76.78%
		**70.66%**	53.27%	99.39%	99.71%	60.75%

	Tayside all residents					
	Tayside ≥65 years	92.22%	67.48%	**99.81%**	99.97%	77.93%
Damerau–Levenshtein (**PCmatch**, **CHserv**, **CHname**)	Fife all residents	**84.17%**	69.42%	99.59%	99.82%	**76.08%**
	Fife ≥65 years	89.94%	**83.52%**	**99.83%**	99.91%	**86.61%**
	Tayside all residents	66.52%	65.8%	**99.56%**	**99.57%**	**66.16%**
	Tayside ≥65 years	**93.4%**	**80.49%**	**99.88%**	**99.97%**	**86.46%**
Damerau–Levenshtein (**PCmatch**, **CHtown**, **CHserv**, **CHname**)	Fife all residents	83.96%	64.4%	99.52%	**99.83%**	72.89%
	Fife ≥65 years	89.87%	78.02%	99.78%	99.91%	83.53%
	Tayside all residents	66.28%	64.59%	99.54%	**99.57%**	65.42%
	Tayside ≥65 years	93.33%	79.67%	**99.88%**	**99.97%**	85.96%

Table S2: Results for different filtering approaches at the patient level in the validation set to validate the best configuration calculated using the Damerau-Levenshtein distance with 1-char-length n-gram. Bold numbers show the best result for each metric in each health board and population.

Method	Health board and data subset	PPV population	Sensitivity population	NPV population	Specificity population	F1 population
Damerau–Levenshtein (no filter)	Fife all residents	57.88%	51.63%	99.63%	99.71%	54.58%
	Fife ≥65 years	90.49%	63.32%	98.95%	99.81%	74.5%
	Tayside all residents	42.49%	75.57%	99.84%	99.33%	54.4%
	Tayside ≥65 years	89.1%	65.72%	99.08%	99.78%	75.65%
Damerau–Levenshtein (**PCmatch**)	Fife all residents	83.48%	76.15%	99.82%	99.89%	79.65%
	Fife ≥65 years	94.21%	82.9%	99.51%	99.85%	88.19%
	Tayside all residents	48.32%	85.58%	99.9%	99.4%	61.76%
	Tayside ≥65 years	96.34%	80.85%	99.48%	99.92%	87.92%
Damerau–Levenshtein (**PCmatch**, **CHtown**)	Fife all residents	81.95%	64.34%	99.73%	99.89%	72.09%
	Fife ≥65 years	93.7%	71.8%	99.19%	99.86%	81.3%
	Tayside all residents	48.71%	85.46%	99.9%	99.41%	62.05%
	Tayside ≥65 years	95.47%	84.63%	99.59%	99.89%	89.72%

Damerau–Levenshtein (**PCmatch**, **CHserv**)	Fife all residents	70.97%	82.23%	99.86%	99.74%	76.19%
	Fife ≥65 years	96.38%	80.03%	99.43%	99.91%	87.45%
	Tayside all residents	65.74%	82.12%	99.88%	99.72%	73.03%
	Tayside ≥65 years	98.4%	80.02%	**99.46%**	99.96%	88.27%
Damerau–Levenshtein (**PCmatch**, **CHserv**, **CHname**)	Fife all residents	91.27%	88.19%	99.91%	**99.94%**	**89.7%**
	Fife ≥65 years	96.83%	**91.64%**	**99.76%**	99.91%	**94.16%**
	Tayside all residents	68.19%	92.49%	99.95%	**99.95%**	78.5%
	Tayside ≥65 years	98.02%	**93.85%**	**99.83%**	**99.95%**	**95.89%**
Damerau–Levenshtein (**PCmatch**, **CHtown**, **CHserv**, **CHname**)	Fife all residents	**91.43%**	84.03%	99.88%	**99.94%**	87.57%
	Fife ≥65 years	96.98%	88.12%	99.66%	99.92%	92.34%
	Tayside all residents	**68.25%**	92.49%	99.95%	99.72%	**78.54%**
	Tayside ≥65 years	98.02%	93.5%	99.82%	**99.95%**	95.7%

Table S3: Results for different char n-gram lengths at the address level in the validation set to validate the best configuration calculated using the Damerau-Levenshtein distance. Bold numbers show the best result for each metric in each health board and population.

Method	Health board and data subset	PPV addresses	Sensitivity addresses	NPV addresses	Specificity addresses	F1 addresses
Damerau–Levenshtein (1 char)	Fife all residents	**84.17%**	69.42%	99.59%	**99.82%**	**76.08%**
	Fife ≥65 years	89.94%	**83.52%**	**99.83%**	99.91%	86.61%
	Tayside all residents	66.52%	**65.8%**	**99.56%**	99.57%	66.16%
	Tayside ≥65 years	93.4%	80.49%	99.88%	**99.97%**	86.46%
Damerau–Levenshtein (2 chars)	Fife all residents	79.97%	**71.34%**	**99.61%**	99.76%	75.41%
	Fife ≥65 years	**96.62%**	78.57%	99.78%	**99.97%**	**86.67%**
	Tayside all residents	**69.94%**	62.87%	99.52%	**99.65%**	**66.22%**
	Tayside ≥65 years	**94.39%**	**82.11%**	**99.89%**	**99.97%**	**87.83%**

Table S4: Results for different char n-gram lengths at the patient level in the validation set to validate the best configuration calculated using the Damerau-Levenshtein distance. Bold numbers show the best result for each metric in each health board and population.

Method	Health board and data subset	PPV population	Sensitivity population	NPV population	Specificity population	F1 population
Damerau–Levenshtein (1 char)	Fife all residents	**91.27%**	88.19%	**99.91%**	**99.94%**	**89.7%**
	Fife ≥65 years	96.83%	**91.64%**	**99.76%**	99.91%	94.16%
	Tayside all residents	68.19%	**92.49%**	**99.95%**	99.72%	78.5%
	Tayside ≥65 years	98.02%	**93.85%**	**99.83%**	**99.95%**	**95.89%**
Damerau–Levenshtein (2 chars)	Fife all residents	83%	**88.41%**	**99.91%**	99.86%	85.62%
	Fife ≥65 years	**98.99%**	89.82%	99.71%	**99.97%**	**94.18%**
	Tayside all residents	**73.12%**	91.78%	**99.95%**	**99.78%**	**81.4%**
	Tayside ≥65 years	**98.14%**	93.74%	**99.83%**	**99.95%**	**95.89%**

Table S5: Results for different char n-gram lengths at the address level in the validation set to validate the best configuration calculated using the Cosine distance. Bold numbers show the best result for each metric in each health board and population.

Method	Health board and data subset	PPV addresses	Sensitivity addresses	NPV addresses	Specificity addresses	F1 addresses
Cosine (1 char)	Fife all residents	**86.15%**	73.95%	99.65%	**99.84%**	**79.59%**
	Fife ≥65 years	92.64%	**82.97%**	**99.83%**	99.93%	**87.54%**
	Tayside all residents	72.9%	66.88%	**99.57%**	99.68%	69.76%
	Tayside ≥65 years	92.59%	81.3%	**99.89%**	99.96%	86.58%
Cosine (2 chars)	Fife all residents	85.36%	70.93%	99.61%	**99.84%**	77.48%
	Fife ≥65 years	92.59%	82.42%	99.82%	99.93%	87.21%
	Tayside all residents	**77.05%**	64.02%	99.53%	**99.75%**	**69.93%**
	Tayside ≥65 years	95.28%	**82.11%**	**99.89%**	**99.98%**	**88.21%**
Cosine (1, 2 chars)	Fife all residents	83.13%	**75.88%**	**99.67%**	99.79%	79.34%
	Fife ≥65 years	**93.67%**	81.32%	99.81%	**99.94%**	87.06%
	Tayside all residents	72.07%	**67.26%**	**99.57%**	99.66%	69.58%
	Tayside ≥65 years	**96.15%**	81.3%	**99.89%**	**99.98%**	88.11%

Table S6: Results for different char n-gram lengths at the patient level in the validation set to validate the best configuration calculated using the Cosine distance. Bold numbers show the best result for each metric in each health board and population.

Method	Health board and data subset	PPV population	Sensitivity population	NPV population	Specificity population	F1 population
Cosine (1 char)	Fife all residents	**92.75%**	89.2%	99.92%	**99.95%**	**90.94%**
	Fife ≥65 years	97.65%	**92.17%**	**99.78%**	**99.94%**	**94.83%**
	Tayside all residents	83.95%	91.66%	**99.95%**	99.88%	87.64%
	Tayside ≥65 years	97.98%	91.73%	99.78%	99.95%	94.75%
Cosine (2 chars)	Fife all residents	92.59%	88.53%	99.91%	**99.95%**	90.51%
	Fife ≥65 years	97.77%	91.64%	99.76%	**99.94%**	94.61%
	Tayside all residents	**93.7%**	90.35%	99.94%	**99.96%**	**91.99%**
	Tayside ≥65 years	98.4%	**94.21%**	**99.84%**	**99.96%**	**96.26%**
Cosine (1, 2 chars)	Fife all residents	88.04%	**90.21%**	**99.93%**	99.91%	89.11%
	Fife ≥65 years	**97.9%**	91.12%	99.75%	**99.94%**	94.39%
	Tayside all residents	81.13%	**92.25%**	**99.95%**	99.86%	86.34%
	Tayside ≥65 years	**98.51%**	93.5%	99.82%	**99.96%**	95.94%

Table S7: Baseline and state-of-the-art methods for detecting care home residents at the address level in the validation set. Cut-off values are in brackets for the Markov and Phonics scores. Bold numbers show the best result for each metric in each health board and population.

Method	Health board and data subset	PPV addresses	Sensitivity addresses	NPV addresses	Specificity addresses	F1 addresses
Postcode Matching	Fife all residents	51.6%	80.0%	99.7%	99.0%	62.7%
	Fife ≥65 years	40.49%	**91.21%**	**99.91%**	98.65%	56.08%
	Tayside all residents	47.66%	**66.05%**	**99.56%**	99.06%	55.37%
	Tayside ≥65 years	34.11%	**95.12%**	**99.97%**	98.91%	50.21%
Markov score (previous study cut-offs)[a]	Fife all residents	61.73%	**87.01%**	**99.82%**	99.27%	72.22%
	Fife ≥65 years	54.97%	**91.21%**	**99.91%**	99.25%	68.6%
	Tayside all residents	59.1%	63.19%	99.52%	99.43%	61.08%
	Tayside ≥65 years	46.98%	82.11%	99.89%	99.45%	59.76%
Markov score (fine tuned for this dataset)[b]	Fife all residents	**77.96%**	77.32%	99.69%	**99.7%**	**77.64%**
	Fife ≥65 years	**90.85%**	76.37%	99.76%	**99.92%**	**82.99%**
	Tayside all residents	**66.47%**	57.22%	99.44%	**99.62%**	**61.5%**
	Tayside ≥65 years	**70%**	79.67%	99.88%	**99.8%**	**74.52%**
Phonics score (previous study cut-offs)[c]	Fife all residents	60.36%	76.49%	99.68%	99.32%	67.47%
	Fife ≥65 years	47.95%	90.11%	99.9%	99.01%	62.6%

	Tayside all residents	51.03%	64.59%	99.54%	99.19%	57.01%
	Tayside ≥65 years	36.36%	94.31%	**99.97%**	99.02%	52.49%
Phonics score (fine-tuned for this dataset)[d]	Fife all residents	60.36%	76.49%	99.68%	99.32%	67.47%
	Fife ≥65 years	47.95%	90.11%	99.9%	99.01%	62.6%
	Tayside all residents	51.03%	64.59%	99.54%	99.19%	57.01%
	Tayside ≥65 years	37.8%	89.43%	99.94%	99.13%	53.14%

a. Markov score using the same method and score cut-offs as Burton et al [12] (allocated as care home address when Markov score >29.6 for all data subsets)
b. Markov score using the same methods as Burton e al [12] but cut-offs fine-tuned for this dataset (allocated as care home when Markov score >47 in Fife all, >62 in Fife ≥65, >37 in Tayside all, >45 in Tayside ≥65).
c. Phonics score using the same method and score cut-offs as Burton et al [12] (allocated as care home address when Markov score >13 for all data subsets)
d. Phonics score using the same methods as Burton e al [12] but cut-offs fine-tuned for this dataset (allocated as care home when Markov score >13 in Fife all, >13 in Fife ≥65, >13 in Tayside all, >30 in Tayside ≥65).

Table S8: Baseline and state-of-the-art methods for detecting care home residents at the patient level in the validation set. Cut-off values are in brackets for the Markov and Phonics scores. Bold numbers show the best result for each metric in each health board and population.

Method	Health board and data subset	PPV population	Sensitivity population	NPV population	Specificity population	F1 population
Postcode Matching	Fife all residents	40.98%	**92.8%**	**99.94%**	98.98%	56.86%
	Fife ≥65 years	67.76%	**94.13%**	**99.83%**	98.71%	78.8%
	Tayside all residents	36.38%	94.04%	99.96%	98.92%	52.46%
	Tayside ≥65 years	69.31%	**99.05%**	**99.97%**	98.81%	81.56%
Markov score (previous study cut-offs)[a]	Fife all residents	50.25%	89.54%	99.92%	99.33%	64.38%
	Fife ≥65 years	77.57%	93.47%	99.81%	99.22%	84.78%
	Tayside all residents	59.21%	**94.99%**	**99.97%**	99.57%	72.95%
	Tayside ≥65 years	80.04%	89.6%	99.72%	99.4%	84.55%
Markov score (fine tuned for this dataset)[b]	Fife all residents	**76.99%**	88.08%	99.91%	**99.8%**	**82.16%**
	Fife ≥65 years	**96.7%**	84.2%	99.55%	**99.92%**	**90.02%**
	Tayside all residents	**71.89%**	89.63%	99.93%	**99.77%**	**79.79%**
	Tayside ≥65 years	**91.6%**	86.41%	99.63%	**99.79%**	**88.93%**
Phonics score (previous	Fife all residents	52.58%	90.55%	99.93%	99.38%	66.53%
		74.56%	93.34%	99.81%	99.08%	82.9%

study cut-offs)[c]	Fife ≥65 years					
	Tayside all residents	46%	93.21%	99.96%	99.28%	61.6%
	Tayside ≥65 years	71.86%	98.7%	99.96%	98.95%	83.17%
Phonics score (fine-tuned for this dataset)[d]	Fife all residents	52.58%	90.55%	99.93%	99.38%	66.53%
	Fife ≥65 years	74.56%	93.34%	99.81%	99.08%	82.9%
	Tayside all residents	46%	93.21%	99.96%	99.28%	61.6%
	Tayside ≥65 years	70.53%	82.62%	99.53%	99.07%	76.1%

a. Markov score using the same method and score cut-offs as Burton et al [12] (allocated as care home address when Markov score >29.6 for all data subsets)
b. Markov score using the same methods as Burton e al [12] but cut-offs fine-tuned for this dataset (allocated as care home when Markov score >47 in Fife all, >62 in Fife ≥65, >37 in Tayside all, >45 in Tayside ≥65).
c. Phonics score using the same method and score cut-offs as Burton et al [12] (allocated as care home address when Markov score >13 for all data subsets)
d. Phonics score using the same methods as Burton e al [12] but cut-offs fine-tuned for this dataset (allocated as care home when Markov score >13 in Fife all, >13 in Fife ≥65, >13 in Tayside all, >30 in Tayside ≥65).

Table S9: Final results at the address level in the validation set using different measures of distance for string and vectors with the best configurations (2-char-length n-grams in both distances). The filtering process used was the ***PCmatch***, ***CHserv***, *and* ***CHname****. Bold numbers show the best result for each metric in each health board and population.*

Method	Health board and data subset	PPV addresses	Sensitivity addresses	NPV addresses	Specificity addresses	F1 addresses
Levenshtein	Fife all residents	80.74%	70.86%	99.61%	99.77%	75.48%
	Fife ≥65 years	96.62%	78.57%	99.78%	99.97%	86.67%
	Tayside all residents	69.94%	62.87%	99.52%	99.65%	66.22%
	Tayside ≥65 years	94.39%	82.11%	99.89%	99.97%	87.83%
Damerau–Levenshtein	Fife all residents	84.17%	69.42%	99.59%	99.82%	76.08%
	Fife ≥65 years	89.94%	83.52%	99.83%	99.91%	86.61%
	Tayside all residents	66.52%	65.8%	99.56%	99.57%	66.16%
	Tayside ≥65 years	93.4%	80.49%	99.88%	99.97%	86.46%
Jaro	Fife all residents	83.5%	74.09%	99.65%	99.8%	78.51%
	Fife ≥65 years	91.52%	82.97%	99.83%	99.92%	87.03%
	Tayside all residents	72.82%	65.73%	99.56%	99.68%	69.09%
	Tayside ≥65 years	95.28%	82.11%	99.89%	99.98%	88.21%
Jaro- Winkler	Fife all residents	83.17%	74.71%	99.66%	99.8%	78.71%
	Fife ≥65 years	90.48%	83.52%	99.83%	99.91%	86.86%
		72.06%	66.24%	99.56%	99.67%	69.03%

	Tayside all residents					
	Tayside ≥65 years	**97%**	78.86%	99.87%	**99.99%**	87%
City Block	Fife all residents	79.53%	72.1%	99.62%	99.75%	75.63%
	Fife ≥65 years	96.69%	80.22%	99.8%	99.97%	87.69%
	Tayside all residents	69.56%	64.65%	99.54%	99.63%	67.02%
	Tayside ≥65 years	94.5%	**83.74%**	**99.9%**	99.97%	**88.79%**
Euclidean	Fife all residents	77.15%	72.65%	99.63%	99.71%	74.83%
	Fife ≥65 years	**97.93%**	78.02%	99.78%	**99.98%**	86.85%
	Tayside all residents	59.48%	67.39%	99.58%	99.4%	63.19%
	Tayside ≥65 years	95.24%	81.3%	99.89%	99.98%	87.72%
Cosine	Fife all residents	85.36%	70.93%	99.61%	**99.84%**	77.48%
	Fife ≥65 years	92.59%	82.42%	99.82%	99.93%	87.21%
	Tayside all residents	**77.05%**	64.02%	99.53%	**99.75%**	**69.93%**
	Tayside ≥65 years	95.28%	82.11%	99.89%	99.98%	88.21%
Correlation	Fife all residents	**86.18%**	74.98%	99.66%	99.84%	80.19%
	Fife ≥65 years	94.97%	82.97%	99.83%	99.96%	88.56%
	Tayside all residents	74.87%	65.54%	99.55%	99.71%	69.9%
		95.28%	82.11%	99.89%	99.98%	88.21%

	Tayside ≥65 years					
Jensen–Shannon	Fife all residents	76.68%	75.95%	99.67%	99.69%	76.31%
	Fife ≥65 years	96.18%	82.97%	99.83%	99.97%	**89.09%**
	Tayside all residents	68.81%	64.08%	99.53%	99.62%	66.36%
	Tayside ≥65 years	95.24%	81.3%	99.89%	99.98%	87.72%
Canberra	Fife all residents	79.03%	74.09%	99.65%	99.73%	76.48%
	Fife ≥65 years	95.39%	79.67%	99.8%	99.96%	86.83%
	Tayside all residents	68.26%	67.26%	99.57%	99.59%	67.76%
	Tayside ≥65 years	93.64%	**83.74%**	**99.9%**	99.97%	88.41%
Bray- Curtis	Fife all residents	83.66%	**77.04%**	**99.69%**	99.8%	**80.21%**
	Fife ≥65 years	90.53%	**84.07%**	**99.84%**	99.91%	87.18%
	Tayside all residents	66.79%	**70.44%**	**99.62%**	99.54%	68.56%
	Tayside ≥65 years	92.52%	80.49%	99.88%	99.96%	86.09%

*Table S10: Final results at the patient level in the validation set using different measures of distance for string and vectors with the best configurations (2-char-length n-grams in both distances). The filtering process used was the **PCmatch**, **CHserv**, and **CHname**. Bold numbers show the best result for each metric in each health board and population.*

Method	Health board and data subset	PPV population	Sensitivity population	NPV population	Specificity population	F1 population
Levenshtein	Fife all residents	84.23%	88.3%	99.91%	99.87%	86.22%
	Fife ≥65 years	98.99%	89.82%	99.71%	99.97%	94.18%
	Tayside all residents	73.12%	91.78%	99.95%	99.78%	81.4%
	Tayside ≥65 years	98.14%	93.74%	99.83%	99.95%	95.89%
Damerau–Levenshtein	Fife all residents	91.27%	88.19%	99.91%	99.94%	89.7%
	Fife ≥65 years	96.83%	91.64%	99.76%	99.91%	94.16%
	Tayside all residents	68.19%	92.49%	99.95%	99.95%	78.5%
	Tayside ≥65 years	98.02%	93.85%	99.83%	99.95%	95.89%
Jaro	Fife all residents	89.55%	88.64%	99.91%	99.92%	89.09%
	Fife ≥65 years	97.51%	92.17%	**99.78%**	99.93%	94.77%
	Tayside all residents	84.26%	91.9%	99.95%	99.89%	87.91%
	Tayside ≥65 years	98.39%	93.74%	99.83%	99.96%	96%

Jaro-Winkler	Fife all residents	89.16%	88.86%	99.92%	99.92%	89.01%
	Fife ≥65 years	97.12%	**92.3%**	**99.78%**	99.92%	94.65%
	Tayside all residents	83.99%	91.9%	99.95%	99.88%	87.76%
	Tayside ≥65 years	**98.84%**	90.43%	99.74%	**99.97%**	94.44%
City Block	Fife all residents	82.1%	88.75%	99.91%	99.85%	85.3%
	Fife ≥65 years	99%	90.21%	99.72%	99.97%	94.4%
	Tayside all residents	72.13%	92.85%	99.95%	99.76%	81.19%
	Tayside ≥65 years	98.28%	**94.44%**	**99.85%**	99.96%	**96.32%**
Euclidean	Fife all residents	78.27%	88.75%	99.91%	99.81%	83.18%
	Fife ≥65 years	**99.28%**	89.43%	99.7%	**99.98%**	94.09%
	Tayside all residents	54.12%	**97.14%**	**99.98%**	99.46%	69.51%
	Tayside ≥65 years	98.39%	93.85%	99.83%	99.96%	96.07%
Cosine	Fife all residents	**92.59%**	88.53%	99.91%	**99.95%**	90.51%
	Fife ≥65 years	97.77%	91.64%	99.76%	99.94%	94.61%
	Tayside all residents	**93.7%**	90.35%	99.94%	**99.96%**	**91.99%**

	Tayside ≥65 years	98.4%	94.21%	99.84%	99.96%	96.26%
Correlation	Fife all residents	91.97%	90.21%	**99.93%**	99.94%	**91.08%**
	Fife ≥65 years	98.32%	91.78%	99.76%	99.95%	94.94%
	Tayside all residents	86%	91.54%	99.94%	99.9%	88.68%
	Tayside ≥65 years	98.4%	94.21%	99.84%	99.96%	96.26%
Jensen–Shannon	Fife all residents	77%	89.99%	99.92%	99.8%	82.99%
	Fife ≥65 years	98.73%	91.51%	99.76%	99.97%	**94.99%**
	Tayside all residents	70.11%	92.25%	99.95%	99.74%	79.67%
	Tayside ≥65 years	98.39%	94.09%	99.84%	99.96%	96.19%
Canberra	Fife all residents	81.25%	89.2%	99.92%	99.84%	85.04%
	Fife ≥65 years	98.57%	90.08%	99.72%	99.96%	94.13%
	Tayside all residents	70.18%	92.85%	99.95%	99.74%	79.94%
	Tayside ≥65 years	98.16%	**94.44%**	**99.85%**	99.95%	96.27%
Bray- Curtis	Fife all residents	87.84%	**91%**	**99.93%**	99.9%	89.39%
	Fife ≥65 years	96.72%	**92.3%**	**99.78%**	99.91%	94.46%
		74.24%	92.73%	99.95%	99.79%	82.46%

	Tayside all residents					
	Tayside ≥65 years	97.89%	93.38%	99.82%	99.95%	95.58%

Click here to access/download;Figure;Figure1.tiff

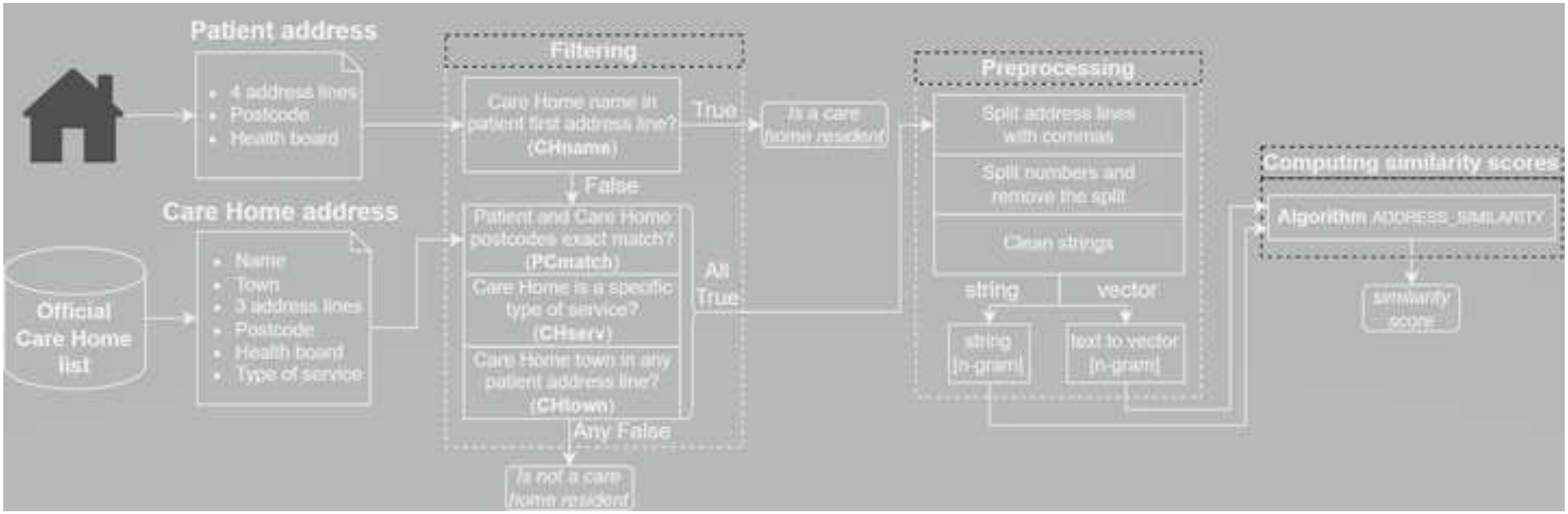

Click here to access/download;Figure;FigureS1.tiff

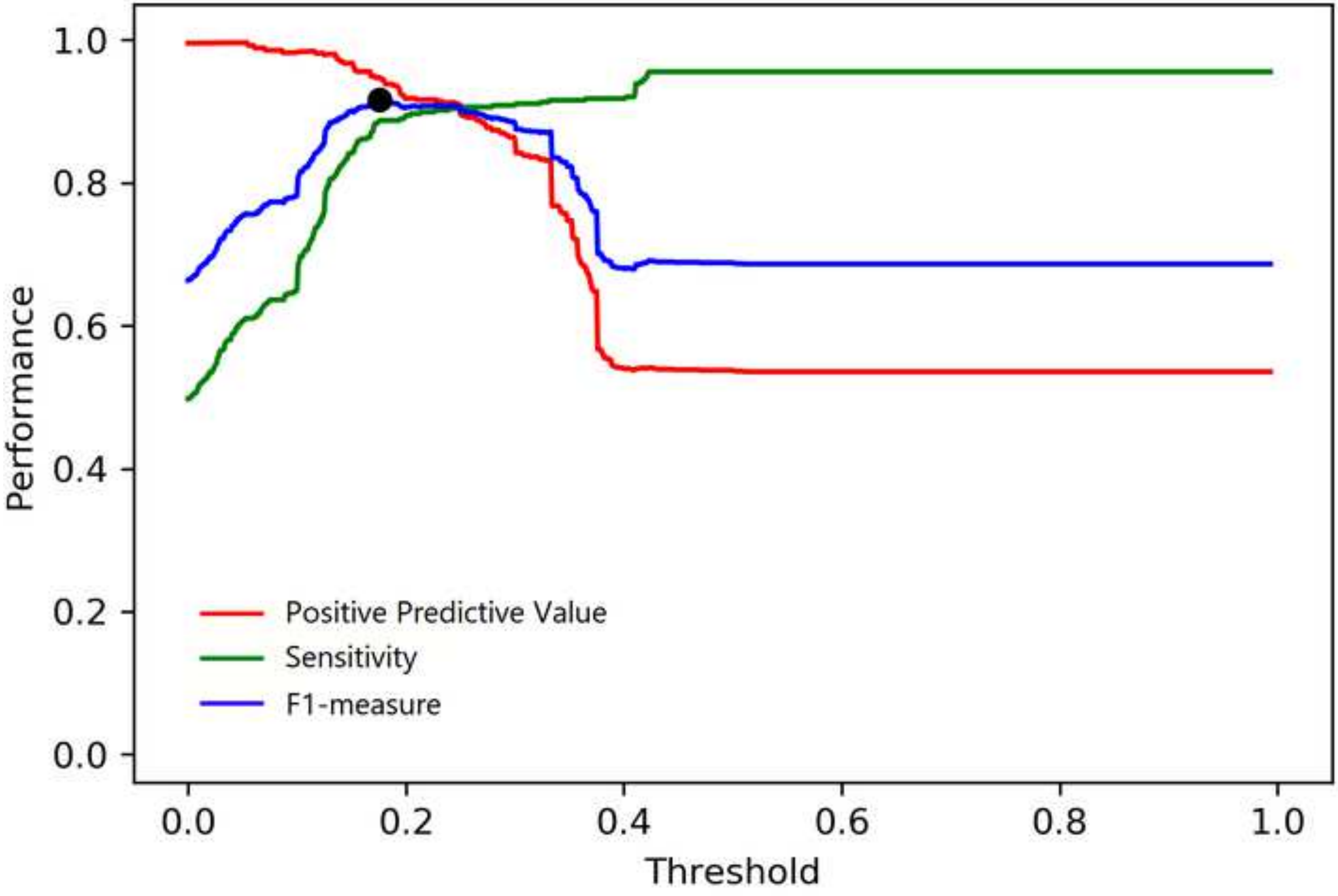

Supplement: Attachment — Submitted filename: PONE-D-23-42367_reviewer.pdf [file pone.0309341.s007.pdf]
