# Supplementary material for: Care home resident identification: A comparison of address matching methods with Natural Language Processing
Source: PLoS One. 2024 Dec 5;19(12):e0309341. doi: 10.1371/journal.pone.0309341 (PMC11620595; doi:10.1371/journal.pone.0309341)
Supplement: Attachment — Submitted filename: Response to Reviewers.docx [file pone.0309341.s008.docx]

**Response to Reviewers**

**Reviewer #1:**

*The article needs further arrangement in a research Journal slandered. you should either expand you background of the study more to include discussion on the main topics embedded in the research title like "Care home resident”, “Natural Language Processing" and so on... Or you include a literature review section before the Methodology with more details.*

*Under the previous study you review also, you need to include details on the methods used by previous researchers, their findings and conclusion of the studies.*

*Under your method, please also include your algorithm from the appendix to fully show your contributions to the method.*

*Your framework (Figure 1) is not very clear also.*

**Response by Authors #1:**

The authors would like to thank the reviewer for the valuable comments and helpful feedback, which have been considered for the revised version of the paper.

The authors have written a completely new and up-to-date section called “Literature review” to expose the background of the current work and the methods used by previous researchers.

In addition, we have aggregated the comparison with previous methods to show the contributions of this new research in the “Comparison with other literature” section.

Moreover, the full algorithm is already made available in the supplementary material, and the authors think that it is best there since only a subset of readers will be interested in reading it. We will of course move it to main text if the editors think that is required.

Furthermore, we have modified Figure 1 to improve the clarity of the proposed method simplifying it and extend the explanation of the system in its caption.

**Reviewer #2:**

*The study used NLP methods (in the preprocess step) for identifying care home residents in primary healthcare data and validated their performance with a manually created ground truth dataset. The paper is a extension of other paper published by some of the here authors.*

*Generally speaking, the paper is well written.*

*Some issues:*

*More information about how the work of the five assessors was executed is required, all five assessors analized all records and a posterior consensus was conduct or a different method was used?*

*The baseline methods names presented in the result tables should be mentioned in the section "Methods" with the same identification.*

*No analysis were presented about NPV and Specificity, even though authors argued that the main objective was to optimize the precision and recall measures (throught the F1), I suggest some analysis over the results or a removal from the paper.*

*The selected method for data split in training and validation (or testing) was the percentage split. Even though it is a applicable method in some contexts, cross-validation is more suitable to ensure that all samples are used at least one time as validation (or testing) data, avoiding a possible bias in the split process.*

*"While previous works had focused on improving the PPV or sensitivity individually, the present study aimed to obtain the best model that can perform well in both scenarios optimizing the F1 metrics which is its harmonic mean"*

*As suggestion, the precision-recall curves are a more suitable method to deal with the tradeoff between the precision (PPV) and recall (sensitivity) measures and provide a more holistic threshold independent evaluation usind the* *Area Under Precision Recal Curve (AUPRC).*

**Response by Authors #2:**

The authors would like to thank the reviewer for the valuable comments and helpful feedback, which have been considered for the revised version of the paper.

The authors have extended the information on how the five assessors labelled all records in the “Reference standard” section.

In addition, the methods presented in the result table are now mentioned in the “Address matching methods” from the Method section.

Moreover, we have removed the NPV and Specificity metrics from the main text, but we leave them in the tables of the supplementary material for future works.

Furthermore, the authors agree that cross-validation is more suitable to avoid bias in the split process, and that the precision-recall curves and the AUPRC add more information about the results. However, most of the previous studies used PPV, NPV, sensitivity, and specificity over a fixed set, so we wanted to do the same as the related works to compare our results with them on a specific test set and show our improvements over these metrics, therefore we decided to do a detailed study in these metrics and take the F1 measure set to select the best model to use in each scenario.
